# I. An Obscure Case of Lameness. II. Tetanus; Recovery

**Published:** 1898-05

**Authors:** W. G. Hollingsworth

**Affiliations:** Utica, N. Y.


### I. AN OBSCURE CASE OF LAMENESS. II. TETANUS; RECOVERY.

By W. G. Hollingsworth, D.V.S.,
UTICA, N. Y.

Case I.—*Subject.* Gray gelding, roadster.

*History.* Slightly lame in the off hindleg for a week. After a careful examination, was unable to make a satisfactory diagnosis, and reserved decision for subsequent visit, which I made one week later, with the same result; animal about the same, and the lesion just as obscure. Had the shoes pulled off and the animal sent to pasture. Occasional visits to the pasture-field during the summer, at which I found the animal much the same. When taking him up in the early fall I made a very thorough examination, using an 8 per cent. solution of cocaine at the hock and fetlock, but he was still lame. At this time a slight swelling was noted at the stifle and bursal; flinch on pressure.

*Diagnosis.* I now came to the conclusion that this was a case of chronic gonitis, although I did not think the lameness was ever well enough marked to indicate any pathologic changes in or around the joint. I aspirated the sac to see if there was any inflammatory lesions existing. Microscopic investigation of the fluid proved negative. Concluded that the pressure of the fluid in the sac was sufficient to produce the slight lameness.

*Treatment.* Applied a blister, and after about four weeks everything seemed to have returned to a normal condition, and I commenced giving light exercise. This was followed by a return of the bursal swelling and lameness. The actual cautery and blister were then applied, and the same restoration obtained, but when exercise was given a return of the swelling and lameness followed, but a longer period intervened. Somewhat discouraged and less hopeful of a good result, I suggested to the owner aspiration and injection into the sac. Injected nearly a drachm of pure carbolic acid, which was followed by a severe inflammation, which subsided in a few days. He was placed in a box-stall for a month, after which he was cautiously driven, the exercise increased as he seemed able to stand it. At this writing there has been no return of bursal swelling, no lameness, and the animal seems absolutely well.

CASE II.—*Subject.* Black mare.

*History* afforded no direct cause; was driven by the owner to my office, stating his belief that his mare had pneumonia, and was apparently well the day prevoius.

*Symptoms:* Jaws were set, nostrils widely dilated; increased respirations, which caused the owner to suspect pulmonary trouble.

*Diagnosis:* Tetanus.

*Prognosis:* Unfavorable; advised removal to my hospital, which was done at 3 P.M. of same day, when the rigidity of muscles was much more marked, general stiffness well pronounced, and mare much excited.

*Treatment:* Placed her in special stall, darkened, and placed slings under her, which seemed to annoy her very much. Gave her 50 c.c. of antitetanus-serum, which dose was repeated early in the morning by an attendant. When visited in the morning the excitement had passed off, otherwise the mare was about the same. Only gruel could be taken, owing to the trismus. The serum treatment was continued in smaller doses, and only carried to 50 c.c., when the animal grew excited or paroxysms were noticed. Trismus continued for two weeks, when the jaws commenced to relax, though the mare continually trying to relax them by grasping the manger. About the eighteenth day she could eat some hay and oats. No purgative medicine was given, as I do not approve of its use. Rectal injections were given to relax the bowels. I try to keep them eating or drinking. She was kept in slings three weeks and then placed in box-stall, 12 x 12; exercised a little daily; was able to get up after lying down without assistance, though the stiffness was still well marked. Exercise continued for a week longer, and then the animal was discharged in good condition.

This is the second case treated with serum, both recovering. The other case was of a more subacute character.

